# Network meta-analysis of treatments for perineal extramammary paget’s disease: Focusing on performance of recurrence prevention

**DOI:** 10.1371/journal.pone.0294152

**Published:** 2023-11-13

**Authors:** Xiaobin Yuan, Ruizhi Xue, Xiaoming Cao

**Affiliations:** 1 Department of Urology, First Hospital of Shanxi Medical University, Taiyuan, Shanxi, China; 2 First College of Clinical Medicine, Shanxi Medical University, Taiyuan, Shanxi, China; University of Turin, S. Anna Hospital, ITALY

## Abstract

**Introduction:**

Perineal extramammary paget’s disease (EMPD) is characterized with high recurrence rate. Although numerous therapeutic measures for this disease have been reported so far, it is unknown whether there is significant difference in their recurrence-preventing efficiency. This study aims to compare the recurrence outcomes of reported perineal EMPD treatments.

**Methods:**

We searched public databases of for published studies concerning perineal EMPD treatments. After screening by inclusion and exclusion criteria, we extracted the data relevant to recurrence rate, and conducted network meta-analysis (NMA) by using Bayesian random-effects approach.

**Results:**

Our analysis included 29 previous studies (involving both male and female patients) and 11 treatment designs which are wide local excision (WLE), local excision (LE), Mohs micrographic surgery (MMS), radiotherapy (RT), radical vulvectomy (RV), photodynamic therapy (PDT), lasers (LS), imiquimod, and WLE+RT, WLE+PDT, WLE+LS. Comparing with WLE, the MMS showed significant advantage in reducing recurrence [OR: 0.18 (0.03–0.87)], while none of the rest treatments has statistically significant results. After removing outlier studies, MMS still has the significant advantages [OR: 0.35 (0.11–0.82)], and LE turned to be the treatment with worst performance [OR: 13 (2.50–110)]. Covariance analysis of follow-up length, gender differences, and lesion locations indicated only short follow-up time could affect the recurrence statistics, which tend to conceal the real differences. Funnel plot demonstrated there is no significant small study effect.

**Conclusion:**

MMS has the best performance on reducing perineal EMPD recurrence, while LE exhibits the worst capability in such regard. Recurrence-preventing abilities of other treatments have no significant difference between each other.

## Introduction

Extramammary Paget’s disease (EMPD) is a rare refractory cutaneous malignancy, it predominantly affects apocrine gland-bearing skin and occurs preferably in perineal organs such as scrotum, penis and vulva [1–3]. The clinicopathological manifestations of EMPD commonly presents as red scaly lesions accompanied by itchy and painful feelings [1, 4]. Although its progression is indolent and metastasis rarely occurs, EMPD is deemed as a challenging disease due to its stubbornly high recurrence rate [5–7]. Since its first report, the therapeutic approaches of EMPD have been under constant exploration and development. Currently, the documented treatments of EMPD are numerous, diversified, and reported by different studies with outcomes varying from one to each other [4, 8]. However, facing literature in such large quantities, clinicians are struggling to find out which therapy has relatively low recurrence rate and thus is superior to others. To gain better insight into the recurrence-preventing ability of current treatments for EMPD, we here performed a network meta-analysis (NMA) based on previous comparative studies. Through our analysis, we hope to provide reference for clinical decision making of EMPD management and future relevant studies.

## Material and methods

### Literature search

We searched databases of Pubmed, Embase and Google Scholar for studies published in English between January 1980 and July 2023, by using ‘the searching strings of “((Extramammary Paget’s disease) AND (treatments)) AND (recurrence)”. We also examined reference lists from all included studies, sourced studies from known reviews on the topic, and sought input from expert contacts. The study and protocol have been registered in PROSPERO database (CRD42023415693).

### Selection criteria

To be eligible for inclusion, 1) studies must be comparative research of at least two EMPD treatments, or observational studies that documented the information of at least two EMPD treatments; 2) the studies must include the basic demographic information (sample size, age, gender ratio, patient number in each treatment group, follow-up time), and details of recurrence (recurrent patients in each treatment group). 3) the studies should be reported original research, clearly described a clinical intervention, and were in the English language.

Exclusion criteria was set as: 1) studies reporting treatments of EMPD patients with metastasis or accompanied by other malignancy; 2) reviews or studies of case reports; 3) studies with eligible sample size fewer than 10 patients; 4) studies reporting EMPD in axilla and other non-perineum areas; 5) studies which have critical risk of bias assessed by ROBINS-I tool (Risk of Bias in Non-randomized Studies of Interventions) [9].

Searching work was conducted by two reviewers (of XY and RX). They also appraised the risk of bias of the results of all studies that met inclusion criteria. Agreement by consensus was reached on the risk of bias for the results of each study. Disagreements were resolved by discussion with a third reviewer (XC).

### Data extraction

The following data were extracted from the 29 articles: first author, country, publication year, sample size, mean age, gender ratio, location of lesion (genitals or perianal region), follow-up time, treatment, patients in each treatment group, recurrence in each treatment group.

### Statistical analysis

Bayesian random-effects approach with Markov-chain Monte Carlo simulation was carried out for the network meta-analysis. Currently, there are several platforms for conducting NMA, and we adopted R software (version 4.0.3) due to the abundance and flexibility of relevant R packages.

Here, in our Bayesian analysis, the package of *gemtc* [10] and *dmetar* [11] were used. Following the package instruction, we arranged the study data (including study labels, sample size, treatments, and recurrence in each treatment) into arm-based form. Applying these information, *gemtc* established the Bayesian hierarchy model (random-effects model) using a Markov Chain Monte Carlo (MCMC) simulation (simulating parameters were set as: Iterations = 100000, Thinning interval = 10, Number of chains = 4, Sample size per chain = 10000) to obtain the posterior distribution (prior parameters were set as N (0, 10, 000) and σ ∼ Unif(0, 5)) of the odds ratio for the treatments. Convergence of the MCMC was confirmed by using the *gelman*.*plot* and *gelman*.*diag* function from the *coda* package (see in S1 Fig). Network graph, direct/indirect evidence plot, and forest plot were generated by the *gemtc* package function of *network*, *direct*.*evidence*.*plot* (*dmetar* package) and *forest*, respectively. We ranked treatments by using surface under the cumulative ranking (SUCRA) score (which measure the extent of certainty that a treatment is better than another) [12]. Global inconsistency was assessed by comparing deviance information criteria (DIC) statistics between different models [13]. Considering the length of follow-up time may exert influence on recurrence outcome, and differences in gender and location of EMPD may yield different results, we also have conducted subgroup analysis based on follow-up time (≥ 60 months and ˂ 60 months), gender (male and female), and lesion locations (genitals and perianal regions). A comparison adjusted funnel plot was used to assess small study effects, where treatments were ordered by expected novelty. Studies with extreme effects on heterogeneity (outlier studies) were detected by using package *NMAoutlier*.

Summary measures used were odds ratio (OR) with 95% credible interval (CrI), p value < 0.05 was considered as statistically significant. The detailed R code transcript used in this research has been provided in S1 File.

## Results

### Characters of included studies

**Fig 1:**
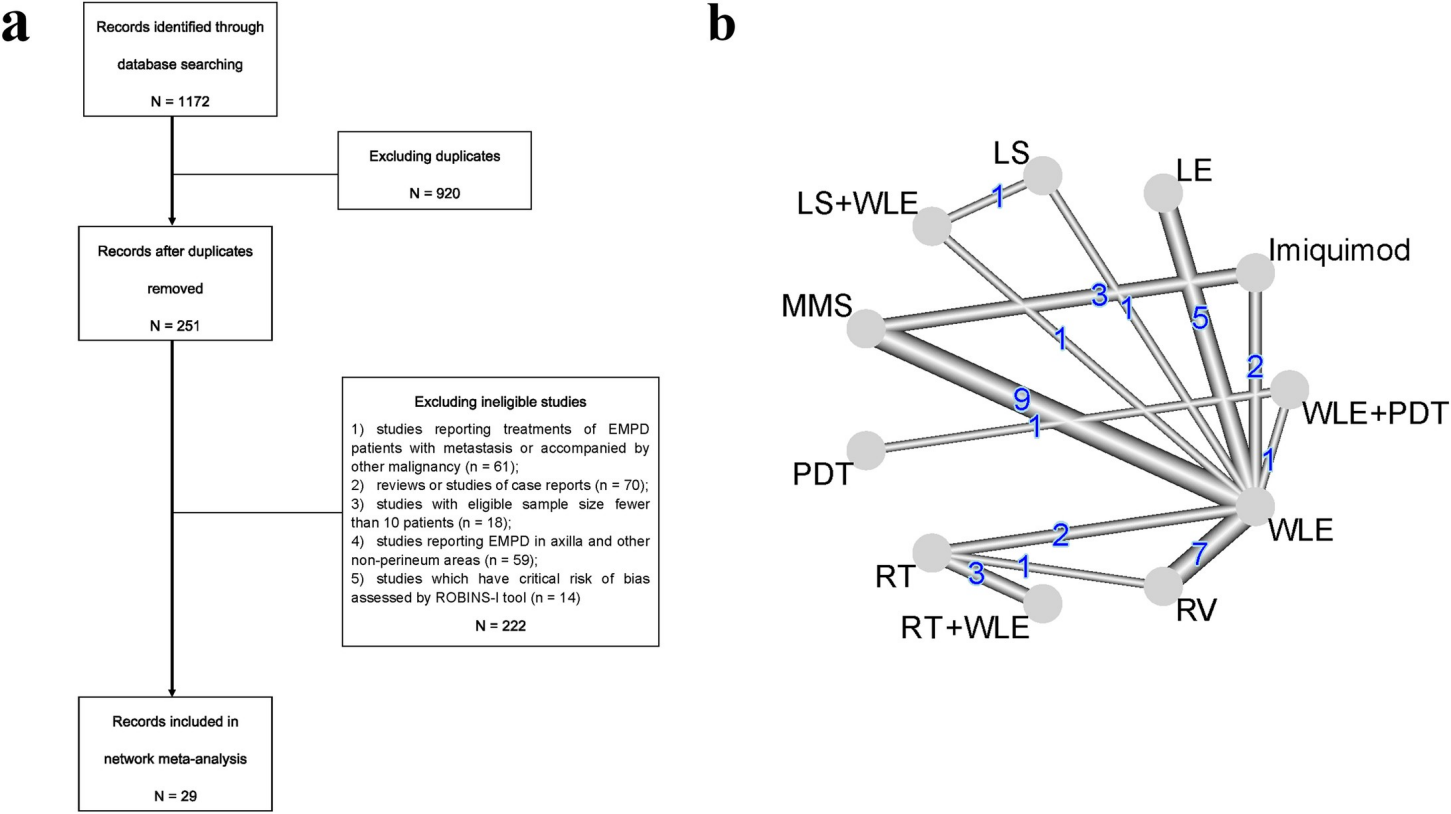
Flow chart of literature selection and network plot of included EMPD treatments. a: Flow chart of the literature selecting process. b: The overall network of EMPD treatments documented by the included studies. Numbers indicate the number of comparative studies of corresponding treatments. Abbreviations: WLE = Wide local excision, LE = Local excision, MMS = Mohs micrographic surgery, RT = Radiotherapy, RV = Radical vulvectomy, PDT = Photodynamic therapy, LS = lasers.

In total, 1172 records were found in our initial searching, the title screening excluded 920 duplicated records, 222 articles were further screened out for unfitting inclusion criteria or with critical risk of bias (see in S1 Table), eventually there are 29 studies left for subsequent analysis (Fig 1A) [14–42]. Among the included studies, 8 therapeutic treatments for EMPD have been identified, which include wide local excision (WLE, with macroscopical surgical margin ≥ 2cm), local excision (LE, with surgical margin < 2cm), Mohs micrographic surgery (MMS), radiotherapy (RT), radical vulvectomy (RV), photodynamic therapy (PDT), imiquimod, and lasers (LS). To be noticed, some of the studies have used combined treatments (which are WLE+RT, WLE+PDT, and WLE+LS) as therapeutic measures for EMPD, and they also have been included in our analysis. Therefore, there are 11 treatment designs in total, the relationship between included studies and treatment designs is demonstrated by network graph (Fig 1B).

The 29 articles come from 7 different countries (United States, United Kingdom, China, Japan, Korea, France, and Italy) with publication year spanning from 1990 to 2023. Among them, the smallest sample size includes 10 patients, while the largest has 278 cases. Mean age varies from 60 to 77 years old, and the median follow-up time is 56.7 months. Detailed information of included studies was presented in Table 1.

**Table 1:** Characters of included studies.

No.	Author	Country	Year	Sample size	Mean age (Year)	Gender ratio (male/female)	Lesion location	Follow-up time (Mon)	Treatments	Patients in each treatment	Recurrence in each treatment
**1**	Long [14]	U.S.	2017	149	70.3	59/90	Genitals (111)	60	WLE	119	40
							Perianal (34)		MMS	30	4
**2**	Lee [15]	Korea	2011	73	67.5	30/43	Genitals (50)	31.5	WLE	57	19
							Perianal (11)		MMS	16	2
**3**	Wang [16]	China	2013	13	75	12/1	Genitals (8)	36	WLE	5	2
							Perianal (1)		WLE+PDT	8	1
**4**	O’Connor [17]	U.S.	2003	95	70.1	45/50	Genitals (80)	59.8	WLE	83	18
							Perianal (15)		MMS	12	2
**5**	Lee [18]	Korea	2009	33	62.8	30/3	Genitals (20)	62.7	WLE	22	8
							Perianal (6)		MMS	11	2
**6**	Gao [19]	China	2015	28	70	28/0	Genitals (24)	12	WLE+PDT	21	9
							Perianal (4)		PDT	7	4
**7**	Nitecki [20]	U.S.	2018	11	67	0/11	Unclear	63.8	RV	6	1
									WLE	5	0
**8**	Wong [21]	China	2016	35	63.9	17/18	Genitals (22)	49	LE	28	16
							Perianal (6)		WLE	7	0
**9**	Hatta [22]	Japan	2008	66	72	55/11	Genitals (62)	70	LE	38	1
							Perianal (4)		WLE	28	4
**10**	Marchesa [23]	U.S.	1997	10	65	5/5	Unclear	70	LE	2	2
									WLE	8	4
**11**	Li [24]	China	2018	278	67.8	104/174	Genitals (178)	78.5	WLE	91	34
							Perianal (25)		MMS	187	3
**12**	Shaco-Levy [25]	U.S.	2010	53	69	0/53	Genitals (43)	66	RV	16	5
							Perianal (10)		WLE	37	11
**13**	Sarmiento [26]	U.S.	1997	11	68.3	5/6	Unclear	78	LE	6	5
									WLE	5	1
**14**	Tebes [27]	U.S.	2002	20	69.1	0/20	Unclear	38.7	RV	5	2
									WLE	15	6
**15**	Curtin [28]	U.S.	1990	29	64	0/29	Unclear	60	RV	25	4
									WLE	4	2
**16**	Louis-Sylvestre [29]	France	2001	49	67.4	0/49	Unclear	24	WLE	29	9
									LS+WLE	14	9
									LS	6	4
**17**	Hegarty [30]	U.S.	2011	13	50–86	8/5	Unclear	48	WLE	8	4
									MMS	5	0
**18**	Isik [31]	U.S.	2016	14	60	4/10	Genitals (0)	60	LE	5	2
							Perianal (14)		WLE	9	3
**19**	Cai [32]	China	2013	43	68.6	0/43	Genitals (43)	54.3	WLE	18	6
							Perianal (0)		RV	17	4
									RT	8	2
**20**	Hata [33]	Japan	2014	41	75	14/27	Genitals (27)	41	RT	24	9
							Perianal (14)		RT+WLE	17	10
**21**	Itonaga [34]	Japan	2014	14	77	7/7	Genitals (11)	71.4	RT	9	5
							Perianal (3)		RT+WLE	5	0
**22**	Hata [35]	Japan	2011	22	72	4/18	Genitals (15)	42	RT	14	13
							Perianal (7)		RT+WLE	8	5
**23**	Kim [36]	U.S.	2017	147	71	65/82	Genitals (71)	30	WLE	124	39
							Perianal (56)		MMS	23	5
**24**	Zollo [37]	U.S.	2000	16	66	8/8	Genitals (14)	70.7	RV	7	2
							Perianal (2)		WLE	9	5
**25**	De Magnis [38]	Italy	2013	34	68.7	0/34	Unclear	76.9	RV	4	3
									WLE	30	12
**26**	Navarrete-Dechent [39]	U.S.	2021	26	71.7	20/6	Genitals (16)	18.3	WLE	21	0
							Perianal (4)		RT	5	2
**27**	Choi [40]	Korea	2021	166	65.4	122/44	Genitals (107)	22.6	WLE	38	9
							Perianal (19)		MMS	114	11
									Imiquimod	14	6
**28**	Christodoulidou [41]	U.K.	2021	10	71.4	10/0	Genitals (8)	76.2	MMS	6	0
							Perianal (2)		Imiquimod	4	2
**29**	Chung [42]	U.S.	2017	42	64.0	23/19	Genitals (25)	36	WLE	20	6
							Perianal (5)		MMS	11	2
									Imiquimod	11	6

Abbreviations: WLE = Wide local excision, LE = Local excision, MMS = Mohs micrographic surgery, RT = Radiotherapy, RV = Radical vulvectomy, PDT = Photodynamic therapy, LS = lasers, CrI = Credible interval.

### Recurrence-preventing ability of different EMPD treatments

**Fig 2:**
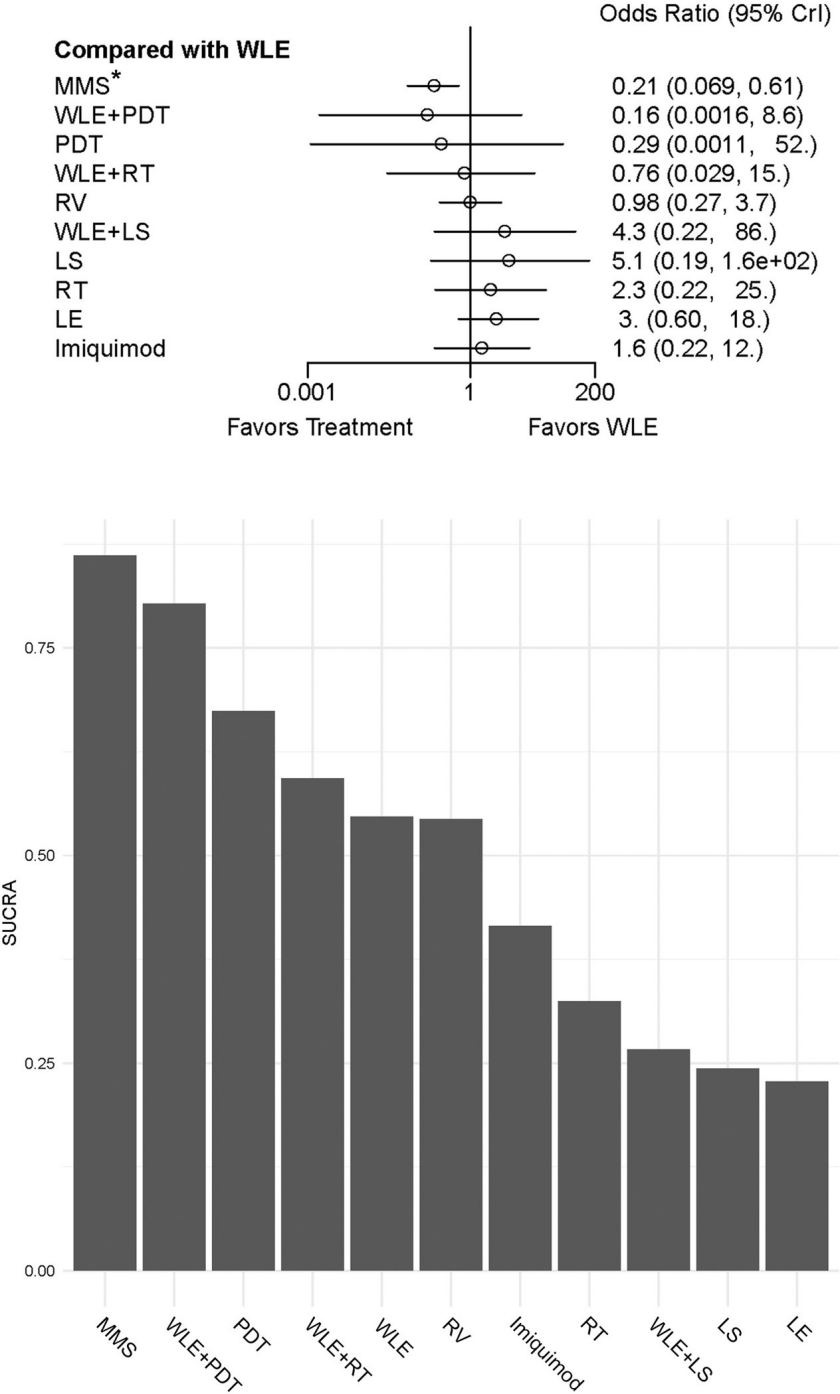
Forest plot and SUCRA score rank of included EMPD treatments concerning their recurrence-prevention abilities. a: Forest plot for recurrence odds ratio of included EMPD treatments (WLE was set as reference treatment). Treatments marked by asterisk show significant difference. b: SUCRA score rank of recurrence-prevention abilities of included EMPD treatments, higher scores indicate better performance on recurrence prevention. Abbreviations: WLE = Wide local excision, LE = Local excision, MMS = Mohs micrographic surgery, RT = Radiotherapy, RV = Radical vulvectomy, PDT = Photodynamic therapy, LS = lasers, CrI = Credible interval, SUCRA = Surface under the cumulative ranking.

During the assessment, the WLE treatment was set as the reference, because this surgery is widely recognized as the most traditional and commonly used treating method for EMPD. Comparing with WLE, only the MMS showed significant difference in reducing EMPD recurrence, with OR of 0.18 (CrI: 0.034 to 0.87), while none of the rest treatments has statistically significant results. The detailed outcome has been demonstrated by forest plot in Fig 2A. The SUCRA ranking results also showed that MMS serves to be the best treatment dealing with EMPD recurrence, while LE has the worst performance in this regard (Fig 2B).

### Outlier studies detection and NMA results after removing them

**Fig 3:**
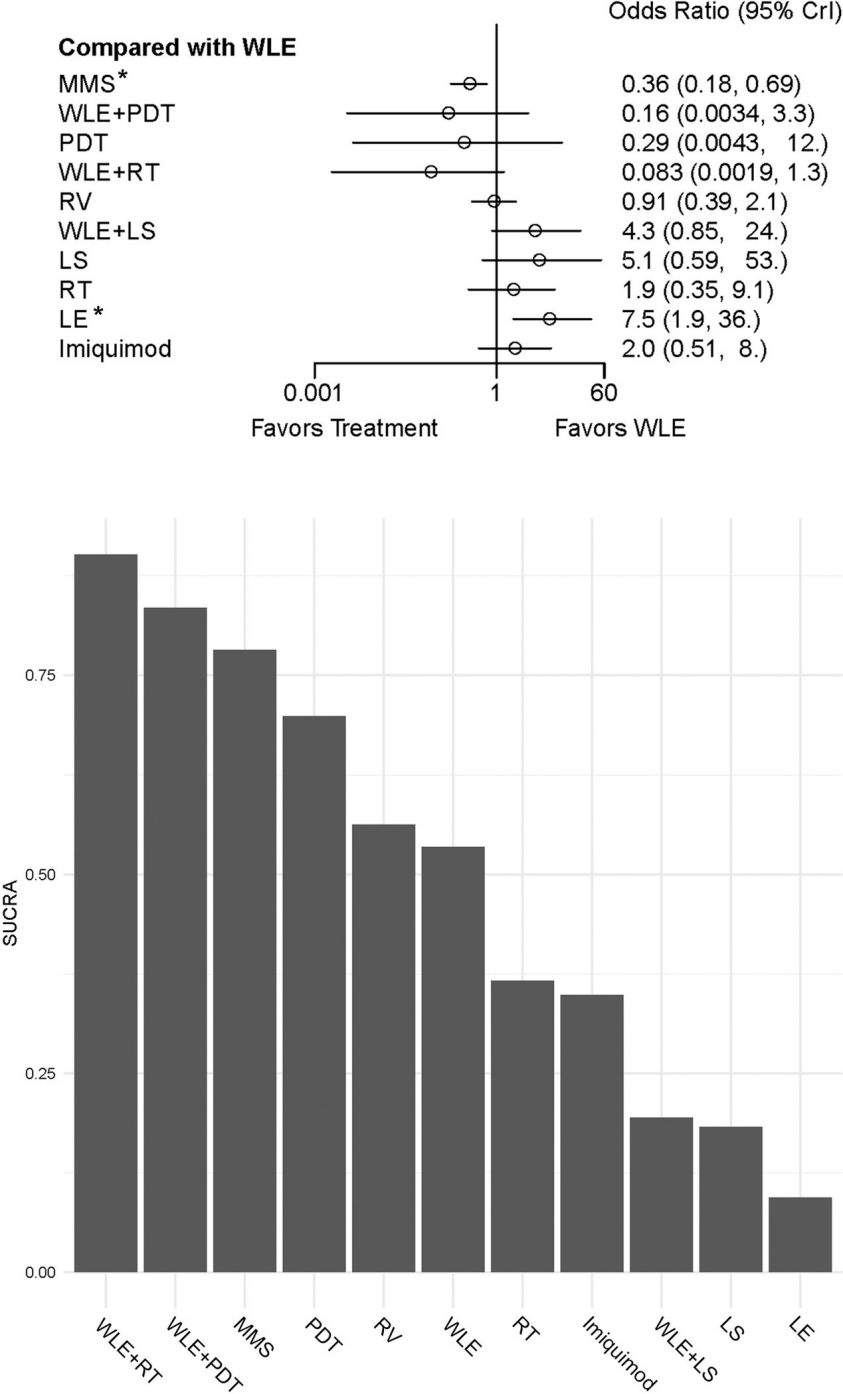
Plot for ratio of variance and DIC score changes. a: The radio of variance contributed by individual studies. Red dots indicate the studies with higher radio than the average level. The unlisted studies were set as initial subset for forward search algorithm according to the instruction of NMAoutlier. b: The DIC scores before and after outlier removal. Abbreviations: DIC = Deviance information criteria.

In the practices of network meta-analysis, some studies might have markedly different characteristics from others, and may exceed the degree of statistical heterogeneity that can be adequately explained by the random-effects model, and these “extreme” studies are usually referred to as the outlier studies [43]. Incorporating outlier studies often causes substantial bias that sometimes can cover up the real difference. To detect the outliers, we used the tailored package of *NMAoutlier* [44]. By adopting forward search algorithm, we have identified 3 major variance-producing outliers, which were demonstrated by the plot for ratio of variance (Fig 3A).

**Fig 4:**
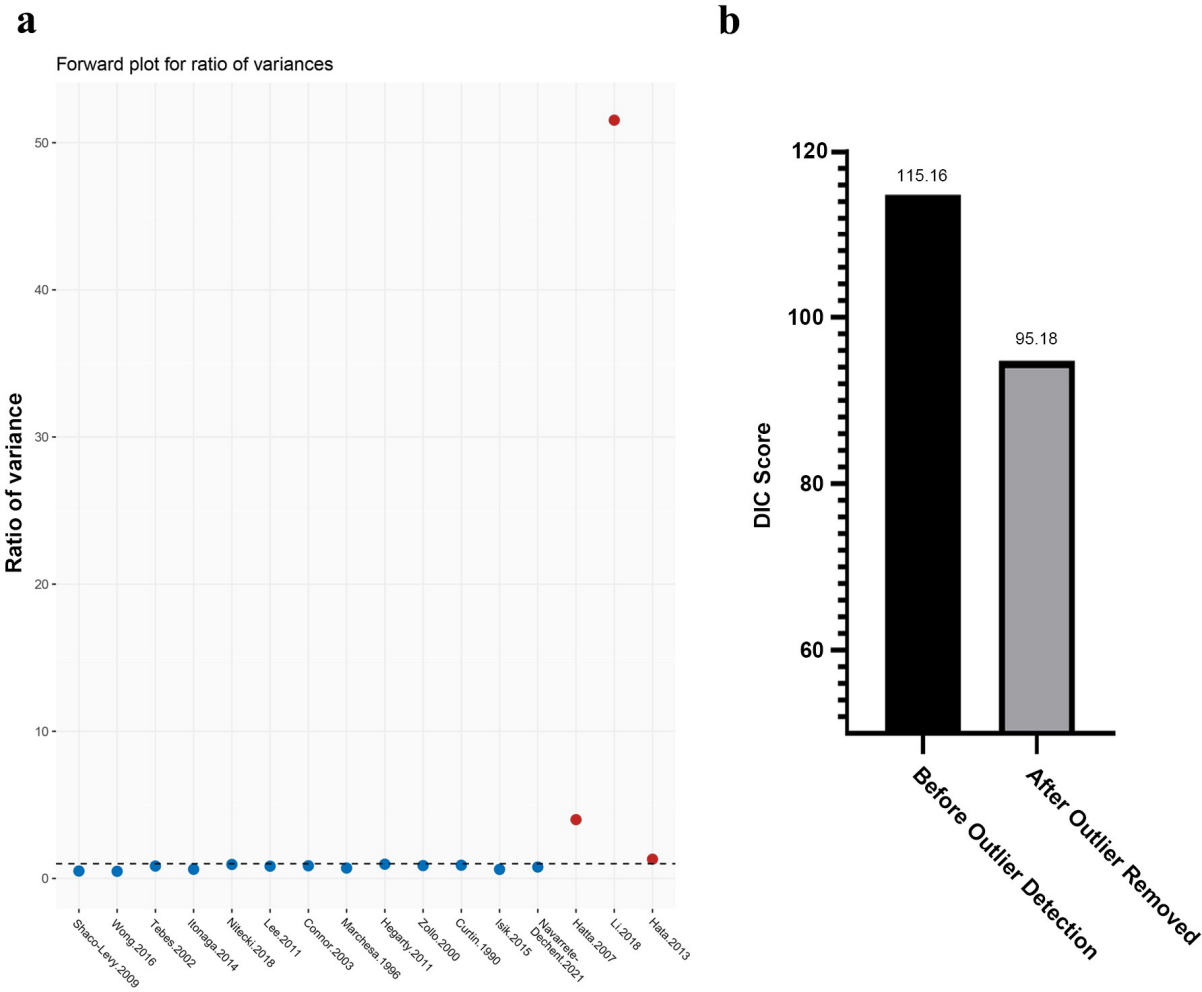
Forest plot and SUCRA score rank of included EMPD treatments after the outlier studies removed. a: Forest plot for recurrence odds ratio of included EMPD treatments (WLE was set as reference treatment). Treatments marked by asterisk show significant difference. b: SUCRA score rank of recurrence-prevention abilities of included EMPD treatments, higher scores indicate better performance on recurrence prevention. Abbreviations: WLE = Wide local excision, LE = Local excision, MMS = Mohs micrographic surgery, RT = Radiotherapy, RV = Radical vulvectomy, PDT = Photodynamic therapy, LS = lasers, CrI = Credible interval, SUCRA = Surface under the cumulative ranking.

After removing the 3 outlier studies, we conducted NMA once again (also setting WLE as reference) for the remained 23 studies. As expected, the DIC score of current Beyasian model is lower, indicating inconsistence of the model is less than the previous one (Fig 3B). The forest plot in this time demonstrated that MMS (OR: 0.35, CrI: 0.11 to 0.82) and LE (OR: 13.0, CrI: 2.5 to 110) have significant difference comparing with WLE treatment in preventing EMPD recurrence (Fig 4A). SUCRA ranking result also has changed, as WLE+RT climbed to the top and followed by WLE+PDT and MMS, while LE remained in the last place (Fig 4B).

### Influence of follow-up length

**Fig 5:**
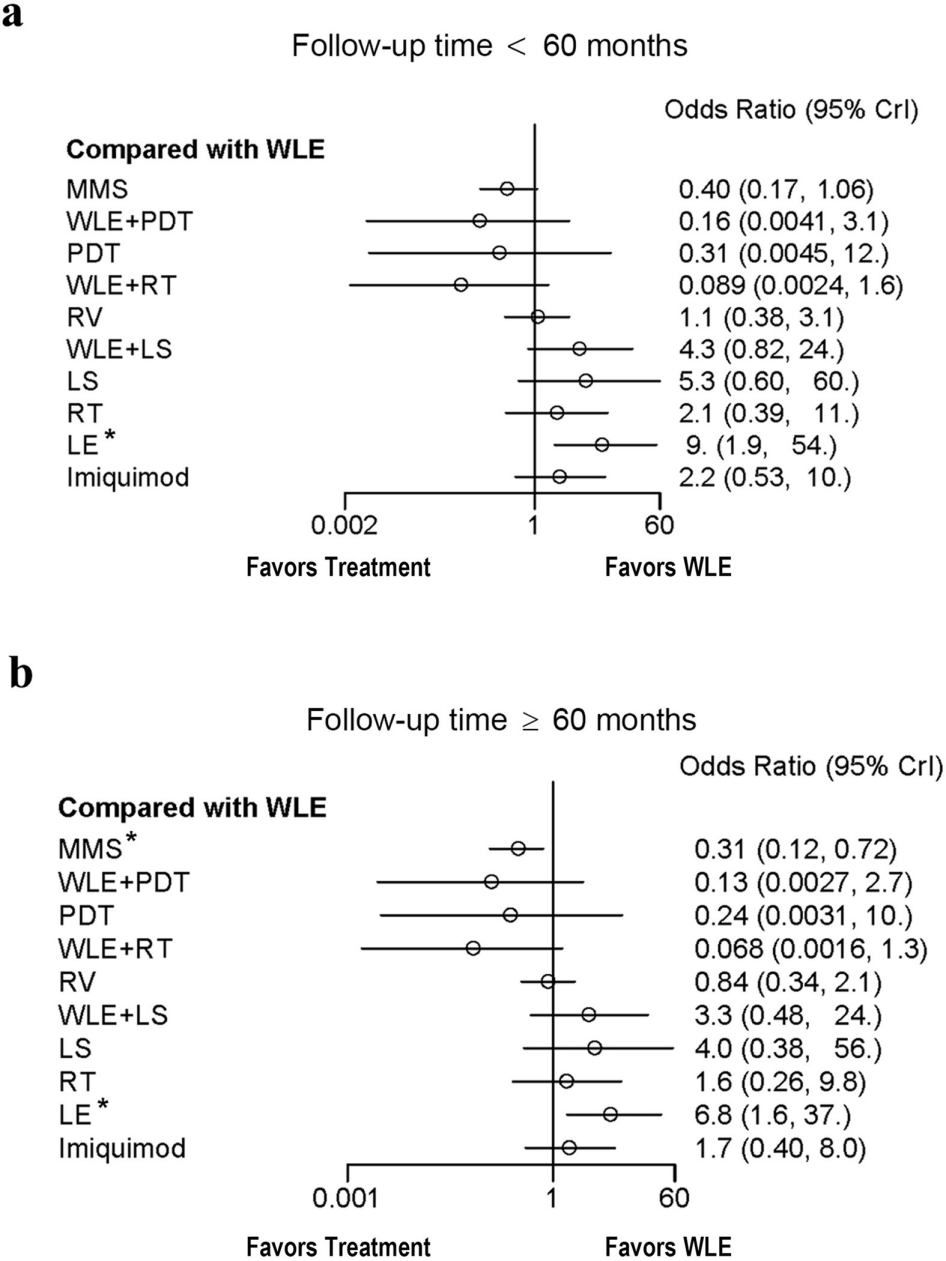
Subgroup analysis of follow-up length influence on EMPD recurrence. a: Forest plot for recurrence odds ratio of included EMPD treatments with follow-up time ˂ 60 months. b: Forest plot for recurrence odds ratio of included EMPD treatments with follow-up time ≥ 60 months. WLE was set as reference treatment, and treatments marked by asterisk show significant difference. Abbreviations: WLE = Wide local excision, LE = Local excision, MMS = Mohs micrographic surgery, RT = Radiotherapy, RV = Radical vulvectomy, PDT = Photodynamic therapy, LS = lasers, CrI = Credible interval.

The variation of covariance from different studies can add heterogeneity of NMA and may impact the magnitude of effect sizes. In our research, we must consider the influence of different follow-up length upon recurrence rates. To check whether such influence exists and causes bias, we divided the outliers-removed studies into two subgroups (follow-up time ≥ 60 months and ˂ 60 months) and used the network meta-regression to conduct subgroup analysis. Results showed that the OR and CI values of the group of longer follow-up time (≥ 60 months) are universally lower than another group (˂ 60 months), presenting a “left-shifting” phenomenon on forest plot (Fig 5). Besides, while LE result is significant in both groups, MMS only has significant result in group of follow-up time ≥ 60 months. Such outcome indicates that follow-up time can influence the effect sizes. If the follow-up length is short (˂ 60 months), it tends to inflate the OR values and cause bias which can cover up the real differences.

### Influence of lesion location and gender differences

**Fig 6:**
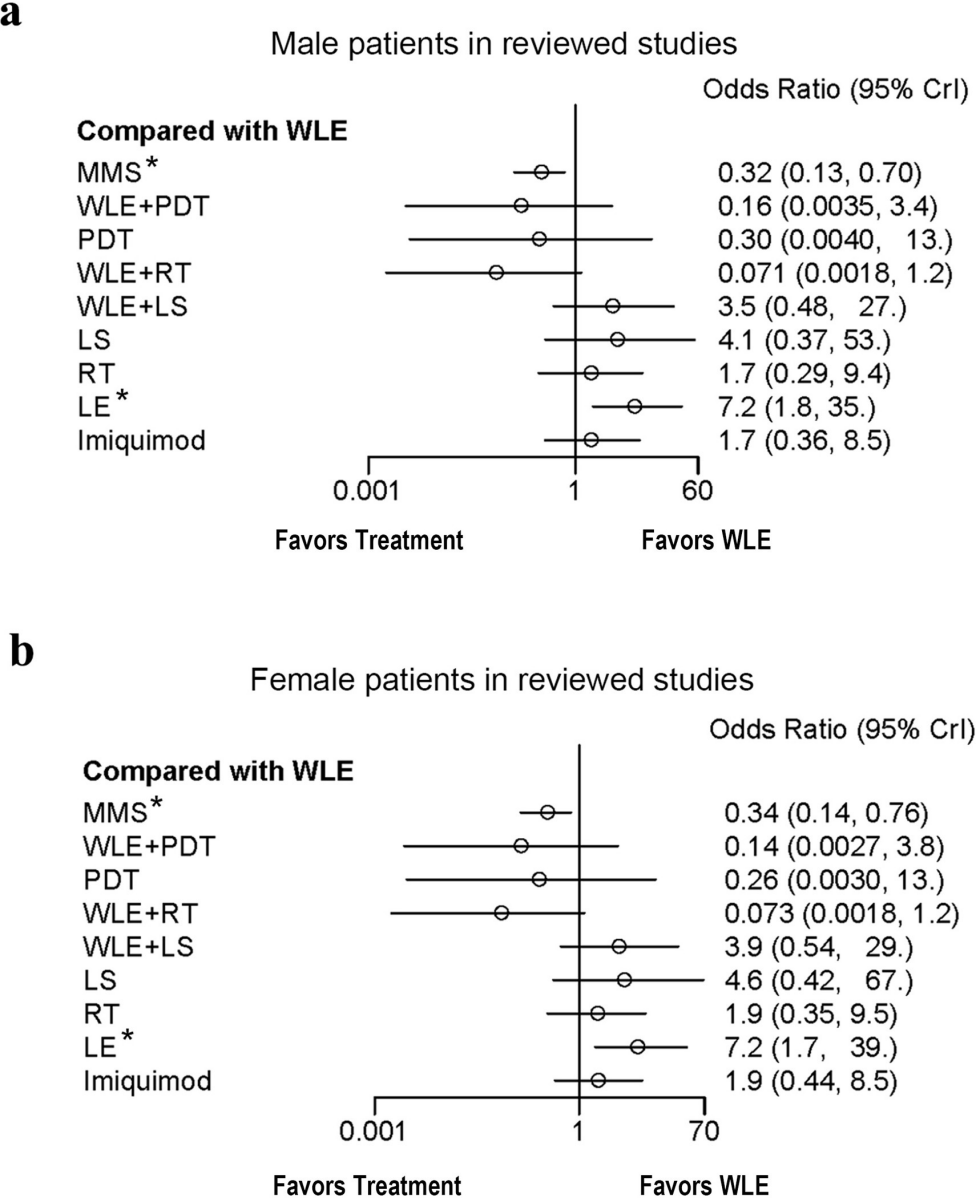
Subgroup analysis of the influence of different gender on EMPD recurrence. a: Forest plot for recurrence odds ratio of EMPD treatments for male patients. b: Forest plot for recurrence odds ratio of EMPD treatments for female patients. WLE was set as the reference treatment, RV was not included as it is solely applied for women. Treatments marked by asterisk show significant difference. Abbreviations: WLE = Wide local excision, LE = Local excision, MMS = Mohs micrographic surgery, RT = Radiotherapy, RV = Radical vulvectomy, PDT = Photodynamic therapy, LS = lasers, CrI = Credible interval.

**Fig 7:**
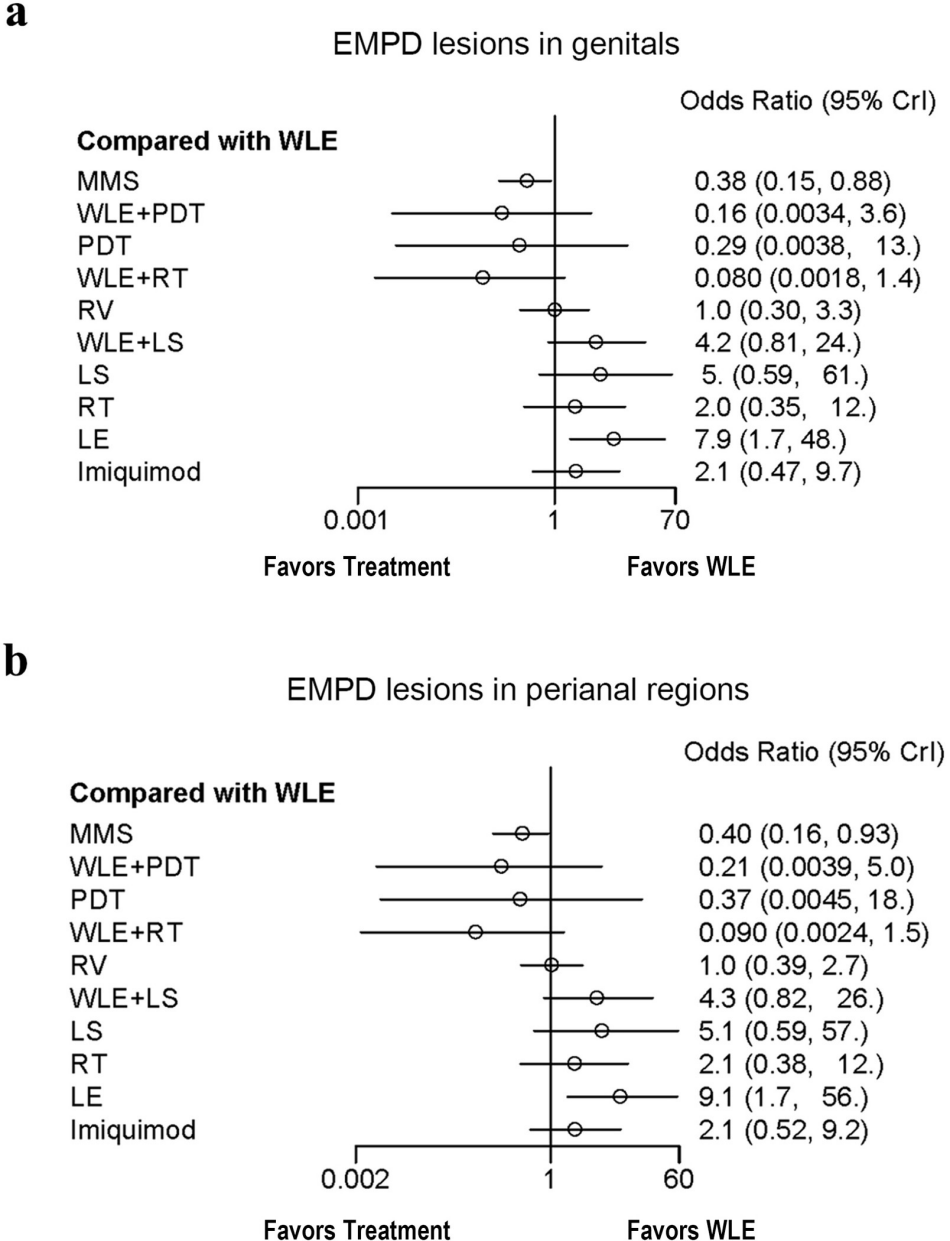
Subgroup analysis of the influence of different lesion locations on EMPD recurrence. a: Forest plot for recurrence odds ratio of EMPD treatments for genital lesions. b: Forest plot for recurrence odds ratio of EMPD treatments for perianal lesions. WLE was set as the reference treatment. Treatments marked by asterisk show significant difference. Abbreviations: WLE = Wide local excision, LE = Local excision, MMS = Mohs micrographic surgery, RT = Radiotherapy, RV = Radical vulvectomy, PDT = Photodynamic therapy, LS = lasers, CrI = Credible interval.

Although the EMPD anatomical sites of our study have been confined to perineum, it still involves many different lesion areas such as penis, scrotum, vulva, crissum skin, etc. To investigate whether different lesion locations could lead to different recurrence outcomes, we categorized the frequently documented perineal EMPD lesion locations into genital region (including penis, scrotum and vulva) and perianal region, then conducted subgroup analysis. Similarly, to rule out the potential influence of gender differences, subgroup analysis of the data respectively from male and female patients has also been carried out (RV is not included in the gender subgroup analysis, because it is solely designed and performed for female patients). According to the forest plot (Figs 6 and 7), gender differences and lesion locations have no significant impact on the final result, as the ranges and values of OR and CI in each group showed little changes. All the plots come to the identical conclusion that MMS has the best, while LE owes the worst, recurrence data compared to WLE.

#### Examine the small study effects by conducting funnel plot

**Fig 8:**
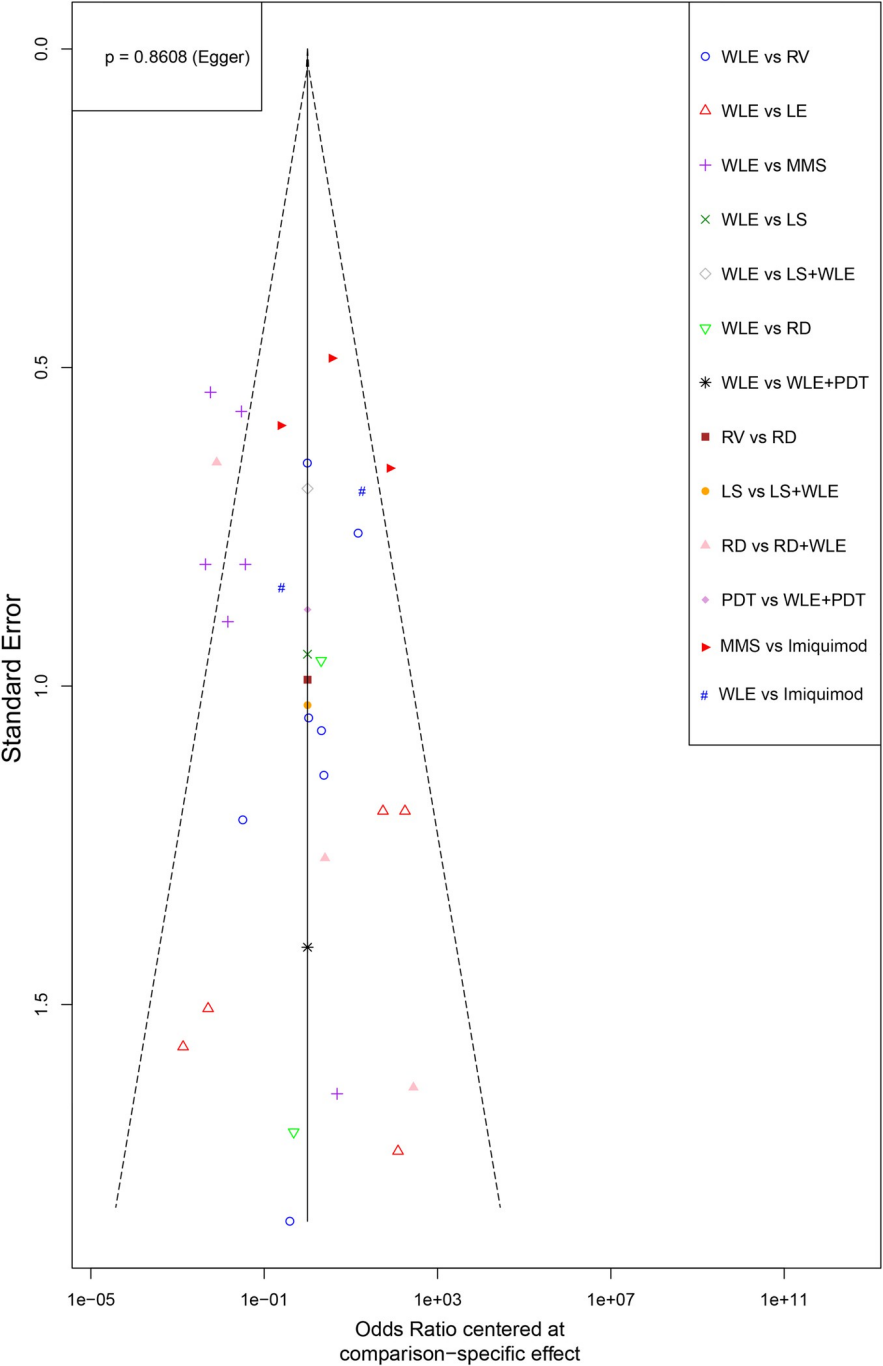
Comparison-adjusted funnel plot. Abbreviations: WLE ‐ Wide local excision, LE = Local excision, MMS = Mohs micrographic surgery, RT = Radiotherapy, RV = Radical vulvectomy, PDT = Photodynamic therapy, LS = lasers, CrI = Credible interval.

Comparison-adjusted funnel plots have been widely proposed to evaluate the risk of small-study effects (equal to publication bias here) in network meta-analyses [12]. Our funnel plot is depicted by *funnel* function of meta package. As shown in Fig 8, the distribution of comparisons is symmetric and p value of Egger’s test is 0.8608, which indicates that the small-study effects are not significant in our research.

## Discussion

This study is focusing on recurrence outcomes of perineal EMPD treatments (for both male and female patients), our literature searching process has identified 11 treatment designs (WLE, LE, MMS, RT, RV, PDT, Imiquimod, LS, WLE+RT, WLE+PDT, and WLE+LS) of perineal EMPD from the 29 included studies. Among them, imiquimod is a topical cream which emerged as a promising therapy recently [3, 45], and the WLE is acknowledged as the most frequently used method for EMPD [1, 4]. Many scholars believe that the residual minimal lesions scattered around the gross tumour edge are the main cause of postoperational recurrence, so a wider resection margin (defined as > 2cm) is proposed by great majority of clinicians [4, 46]. Considering the universality of WLE in EMPD treatments, we set it as the reference in our NMA and hope to find a better alternative.

During the initial analysis of all the 26 included studies, MMS is shown in forest plot as the only treatment that has significant advantages in reducing EMPD recurrence, and it is also recommended by SUCRA rank as the best intervention in such regard. According to literature background, MMS is the surgical procedure which removes cutaneous tumour in a series of horizontal layers. It is performed by removing a thin margin of tissue circumferentially around and deep to the clinical margins of a skin tumor. The specimen is typically removed with a 45-degree bevel to facilitate tissue processing. It is then rapidly frozen and sectioned in a cryostat microtome, allowing for quick tissue processing (about 15 to 30 minutes). Sectioning the tissue in a horizontal direction allows virtually 100% of the tissue margin (peripheral and deep margins) to be examined under the microscope. The process is repeated until the tumor has negative histologic margins [47]. We believe such characters ensure the maximum removal of diseased tissues and thus give the MMS more advantages than WLE, which is relatively rougher in dealing with underlying lesions.

In the following analysis, 3 outlier studies have been detected and heterogeneity of the Beyasian model shows substantial reduction after removing them (DIC score reduced). A closer re-examination of these 3 studies has discovered confounding factors, which include blending unstated treatments into the control groups (Li, et al [24] Hata, et al [33]) and performing preoperative mapping biopsies to unknown number of patients (Hatta, et al [22]). Excluding these confounders is beneficial for bias control, and thus can bring us closer to the real facts.

NMA was conducted once again for the outlier-removed studies. As shown in forest plot, MMS still shows significant advantages in coping EMPD recurrence, while LE in this time, also has significant result which proves it can lead to higher recurrence rates than WLE. This outcome is within our expectation. The range of LE surgical margins is less than 2cm, such resection coverage gives convenience for wound repairing, but also inevitably omits many residual small lesions which are often present in macroscopically normal tissue and bring high risks of recurrence. As an early surgical method for EMPD, LE is rarely reported in recent studies, and clinicians rather adopt flap repairing to achieve the complete resection of EMPD [4, 48].

The SUCRA rank analysis of outlier-removed studies, however, shows inconsistent result with the forest plot, as WLE+RT and WLE+PDT have higher ranking places than MMS. Facing such conflicts, we think the SUCRA ranking result should be interpreted with caution because SUCRA is a mathematical process that calculates the likelihood of becoming the best treatment, it does not consider the magnitude of differences between interventions and its estimates of different treatments often overlap. Besides, only a few included studies focus on the treatments of WLE+PDT and WLE+RT, which associate with indirect comparisons plus low evidence certainty, and this also gives the SUCRA a flaw basis.

In the final parts, we checked the influence of follow-up length, gender difference, lesion locations and small study effect. Usually, different follow-up times tend to yield different recurrence rates, the subgroup analysis has showed that such case exits in our NMA. Comparing with subgroup of longer follow-up length (≥ 60 months), OR values in the shorter follow-up group (< 60 months) have been universally inflated, and the statistical result of MMS becomes no longer significant, which indicates that inadequate follow-up time may conceal the real differences. For the examination of small study effect, funnel plot with Egger’s test is adopted. Results demonstrated that no obvious small study effect was found.

There are some limitations in our NMA. First, we did not conduct subgroup analysis for different pathologic subtypes of EMPD. According to depth of tumour invasion, EMPD is commonly classified into two types which are intraepithelial (tumour cells are confined to the epithelium) and invasive (tumour cells penetrate the dermis) Paget’s diseases. Different extent of infiltration is probably associated with different risks of recurrence [49]. Unfortunately, we have no adequate data source to verify such hypothesis, due to most of the included studies have not documented detailed information of pathologic subtypes. Secondly, the evidence certainty and study samples of some treatments (such as RT, LS, and PDT) are generally insufficient, which affects the reliability of the corresponding results, we hope this situation could be improved with the increase of relevant studies in future. Finally, it is necessary noting that none of the included studies in our NMA is randomized controlled trial and confidence intervals of estimates are generally wide, these may also weaken the evidence basis and compromise the reliability.

In summary, based on included studies our NMA work reveals that, among the listed candidate treatments, MMS has the best performance on reducing perineal EMPD recurrence, while LE exhibits the worst capability in such regard. Recurrence-preventing abilities of other treatments such as RT, LS, WLE and PDT have no significant difference between each other. More relevant comparative studies with high evidence level and detailed pathologic information are required for verifying and furthering above conclusions.

## Supporting information

S1 Checklist PRISMA 2020 checklist. (PDF)

S1 Table Risk of bias appraisal of included studies by ROBINS-I tool. (PDF)

S1 Fig Convergence test of the MCMC (Markov Chain Monte Carlo) simulation. (PDF)

S1 File R code. (PDF)
