# Supplementary material for: Network meta-analysis of treatments for perineal extramammary paget’s disease: Focusing on performance of recurrence prevention
Source: PLoS One. 2023 Nov 13;18(11):e0294152. doi: 10.1371/journal.pone.0294152 (PMC10642846; doi:10.1371/journal.pone.0294152)
Supplement: S1 Table — (PDF) [file pone.0294152.s002.pdf]

Risk of bias appraisal of included studies by ROBINS-I tool.

**Study**	**Confounding (all outcomes)**	**Selection**	**Classification**	**Deviations from intended interventions (assignment)**	**Missing data (all outcomes)**	**Measurement of the outcome (all outcomes)**	**Selection of the reported result (all outcomes)**	**Overall**
Long 2017	Serious	Low	Low	Low	Low	Moderate	Moderate	Serious
Lee 2011	Serious	Low	Low	Low	Serious	Low	Moderate	Serious
Wang 2013	Serious	Low	Low	Low	Moderate	Low	Moderate	Serious
O'Connor 2003	Serious	Low	Low	Low	Serious	Low	Moderate	Serious
Lee 2009	Serious	Low	Low	Low	Low	Moderate	Moderate	Serious
Gao 2015	Serious	Serious	Low	Low	NI	Low	Serious	Serious
Nitecki 2018	Serious	Low	Low	NI	Serious	Low	Moderate	Serious
Bambao 2009	Critical	Low	Low	NI	NI	Serious	Serious	Critical
Li 2011	Critical	Serious	Low	Low	Critical	Serious	Serious	Critical
Wong 2016	Serious	Low	Low	Low	Low	Low	Moderate	Serious
Hatta 2008	Serious	Low	Low	NI	Serious	Low	Moderate	Serious

Bauman 2018	Critical	Low	Low	Low	Critical	Serious	Serious	Critical
Marchesa 1997	Serious	Low	Low	NI	NI	Low	Moderate	Serious
Li 2018	Serious	Low	Low	Low	Serious	Low	Moderate	Serious
Shaco-Levy 2010	Serious	Low	Low	Low	Moderate	Low	Moderate	Serious
Shukla 2016	Critical	Serious	Low	NI	NI	Serious	Critical	Critical
Sarmiento 1997	Serious	Low	Low	Moderate	NI	Low	Moderate	Serious
Tebes 2002	Serious	Moderate	Low	Low	Serious	Low	Moderate	Serious
Curtin 1990	Serious	Low	Low	Low	NI	Low	Low	Serious
Louis-Sylvestre 2001	Serious	Low	Low	Low	Low	Low	Low	Serious
Hegarty 2011	Serious	Low	Low	Low	Low	Low	Moderate	Serious
Isik 2016	Serious	Low	Low	NI	Serious	Low	Moderate	Serious
Cai 2013	Serious	Low	Low	NI	Moderate	Low	Moderate	Serious

Hata 2014	Serious	Low	Low	NI	Low	Low	Moderate	Serious
Itonaga 2014	Serious	2014	Low	Low	NI	Low	Moderate	Serious
Yasar 2015	Critical	Low	Low	Low	NI	Serious	Serious	Critical
Hata 2011	Serious	Moderate	Low	Low	Serious	Low	Moderate	Serious
Minicozzi 2010	Critical	Serious	Low	Low	Moderate	Serious	Critical	Critical
Kim 2017	Serious	Low	Low	Serious	Low	Low	Moderate	Serious
Zollo 2000	Serious	Low	Low	Low	Low	Low	Moderate	Serious
De Magnis 2013	Serious	Low	Low	Low	Serious	Low	Moderate	Serious
Navarrete-Dechent 2021	Serious	Low	Low	Serious	Serious	Low	Moderate	Serious
Choi 2021	Serious	Moderate	Low	Low	Serious	Low	Moderate	Serious
Christodou-lidou 2021	Serious	Low	Low	Low	Serious	Low	Moderate	Serious
Chung 2017	Serious	Low	Low	Low	NI	Low	Low	Serious

Abbreviation: NI = No information.
