# Supplementary material for: Network meta-analysis of treatments for perineal extramammary paget’s disease: Focusing on performance of recurrence prevention
Source: PLoS One. 2023 Nov 13;18(11):e0294152. doi: 10.1371/journal.pone.0294152 (PMC10642846; doi:10.1371/journal.pone.0294152)
Supplement: S1 Fig — (PDF) [file pone.0294152.s003.pdf]

Convergence test of the MCMC (Markov Chain Monte Carlo) simulation

1) *gelman.plot* results:

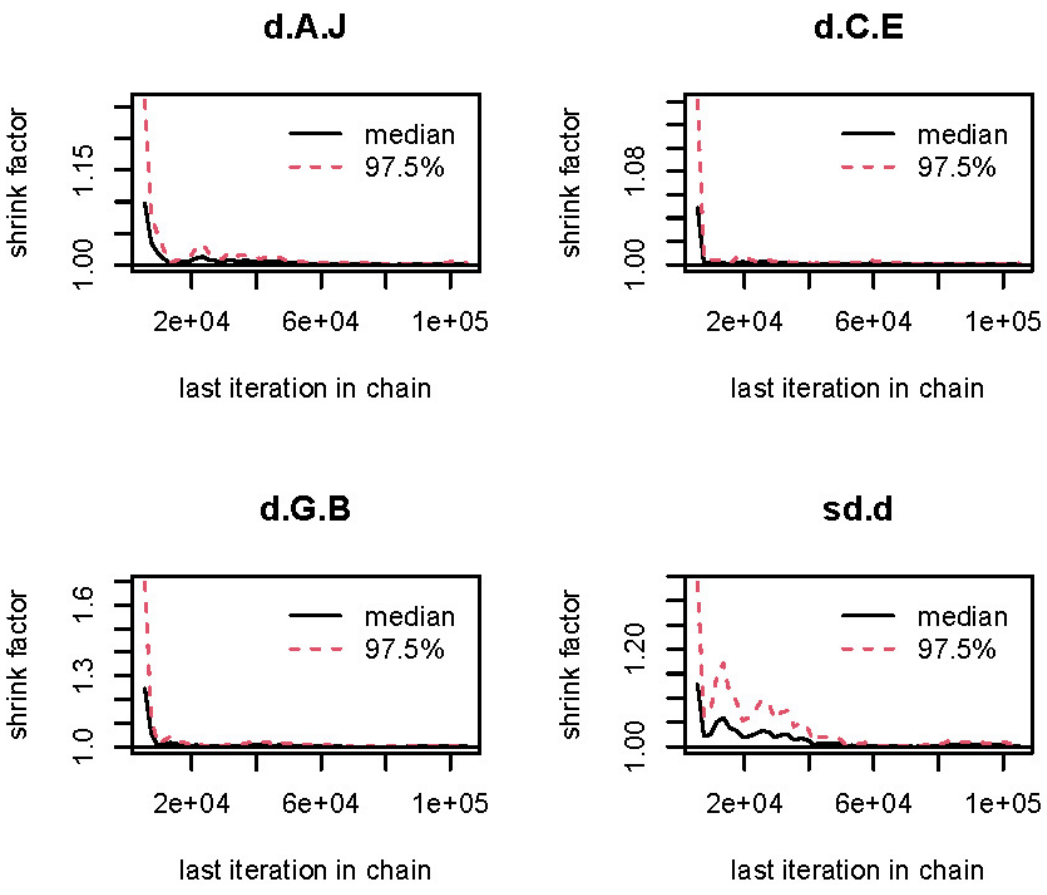

2) *gelman.diag* results:

Potential scale reduction factors:

	Point est.	Upper C.I.
d.A.C	1	1
d.A.D	1	1
d.A.F	1	1
d.A.G	1	1
d.A.H	1	1
d.A.I	1	1
d.A.J	1	1
d.C.E	1	1
d.G.B	1	1
sd.d	1	1

Multivariate psrf: 1

We have tested the convergence of the MCMC by using the *gelman.plot* and *gelman.diag* function (Brooks-Gelman-Rubin method) from the *coda* package. By calculating the shrink factor at several points in time, *gelman.plot* shows the shrink factor has really converged as the it stops fluctuating.

The psrf (potential scale reduction factor proposed by Gelman and Rubin) is a statistics that compares the within-chain and between-chain variances and values smaller than 1.1 indicate convergence.
