# Supplementary material for: Network meta-analysis of treatments for perineal extramammary paget’s disease: Focusing on performance of recurrence prevention
Source: PLoS One. 2023 Nov 13;18(11):e0294152. doi: 10.1371/journal.pone.0294152 (PMC10642846; doi:10.1371/journal.pone.0294152)
Supplement: S1 File — (PDF) [file pone.0294152.s004.pdf]

# R code:

```
treatments<- read.csv("~meta/Bayesian/data/treatments outlier removed.csv",header=T,sep = ",",skip=0,row.names = 1)
data<- read.csv("~meta/Bayesian/data/outlierremoved.csv",header=T,sep = ",",skip=0,row.names = 1)

library(gemtc)

network<- mtc.network(data.ab = data, treatments=treatments)

print(network)
summary(network)

plot(network,
     use.description = TRUE)
plot(network,
     use.description = TRUE,               # Use full treatment names
     vertex.color = "white",               # node color
     vertex.label.color = "gray10",        # treatment label color
     vertex.shape = "sphere",              # shape of the node
     vertex.label.family = "Helvetica", # label font
     vertex.size = 16,                     # size of the node
     vertex.label.dist = 2,                # distance label-node center
     vertex.label.cex = 1.5,               # node label size
     edge.curved = 0.2,                    # edge curvature
     layout = layout.fruchterman.reingold)

model <- mtc.model(network,
                   likelihood = "binom",
                   link = "logit",
                   linearModel = "random",
                   n.chain = 4)

mcmc2 <- mtc.run(model, n.adapt = 5000, n.iter = 1e5, thin = 10)

mcmc2
summary(mcmc2)
summary(mcmc2)$DIC

gelman.plot(mcmc2)

gelman.diag(mcmc2)
```

```
nodesplit <- mtc.nodesplit(network,
                           linearModel = "random",
                           likelihood = "binom",
                           link = "logit",
                           n.adapt = 5000,
                           n.iter = 1e5, thin = 10)



plot(network)

summary(network)

summary(nodesplit)

plot(summary(nodesplit))

library(dmetar)
rank.probability <- rank.probability(mcmc2)
sucra <- dmetar::sucra(rank.probability, lower.is.better = TRUE)
sucra
plot(sucra)


rank <- rank.probability(mcmc2, preferredDirection = -1)
rank
plot(rank, beside=TRUE)

tbl <- relative.effect.table(mcmc2, "A")

print(tbl)

forest(relative.effect(mcmc2, t1 = "A"),
       use.description = TRUE,
       left.label="Favors Treatment", right.label="Favors WLE", center.label=NULL,
       ask=dev.interactive(orNone=TRUE),
       draw.no.effect=TRUE) # Use long treatment names


#subgroup analysis
study.info <- read.csv("~meta/Bayesian/study.info.csv",header=T,sep = ",",skip=0,row.names
= 1)
network.mr <- mtc.network(data.ab = data,
                          studies = study.info,
```

```
                                    treatments = treatments)

regressor <- list(coefficient = "shared",
                  variable = "following.up.time",
                  control = "A")

model.mr <- mtc.model(network.mr,
                      likelihood = "binom",
                      link = "logit",
                      type = "regression",
                      regressor = regressor)

mcmc3 <- mtc.run(model.mr,
                 n.adapt = 5000,
                 n.iter = 1e5,
                 thin = 10)

summary(mcmc3)

forest(relative.effect(mcmc3, t1 = "A", covariate = 1),
       use.description = TRUE)
title("Follow-up time ≥ 60 months")

forest(relative.effect(mcmc3, t1 = "A", covariate = 0),
       use.description = TRUE)
title("Follow-up time < 60 months")
```
